# Advances in Rare‐Earth Tritelluride Quantum Materials: Structure, Properties, and Synthesis

**DOI:** 10.1002/advs.202004762

**Published:** 2021-03-19

**Authors:** Kentaro Yumigeta, Ying Qin, Han Li, Mark Blei, Yashika Attarde, Cameron Kopas, Sefaattin Tongay

**Affiliations:** ^1^ School for Engineering of Matter Transport and Energy Arizona State University Tempe AZ 85287 USA

**Keywords:** charge density waves, rare‐earth tritellurides, superconductivity

## Abstract

A distinct class of 2D layered quantum materials with the chemical formula of *R*Te_3_ (*R* = lanthanide) has gained significant attention owing to the occurrence of collective quantum states, superconductivity, charge density waves (CDW), spin density waves, and other advanced quantum properties. To study the Fermi surface nesting driven CDW formation, the layered *R*Te_3_ family stages an excellent low dimensional genre system. In addition to the primary energy gap feature observed at higher energy, optical spectroscopy study on some *R*Te_3_ evidence a second CDW energy gap structure indicating the occurrence of multiple CDW ordering even with light and intermediate *R*Te_3_ compounds. Here, a comprehensive review of the fundamentals of *R*Te_3_ layered tritelluride materials is presented with a special focus on the recent advances made in electronic structure, CDW transition, superconductivity, magnetic properties of these unique quantum materials. A detailed description of successful synthesis routes including the flux method, self‐flux method, and CVT along with potential applications is summarized.

## Graphical abstract

A distinct class of 2D layered quantum materials with the chemical formula of *R*Te_3_ (*R* = lanthanide) has gained significant attention owing to the occurrence of quantum properties. This review focuses the fundamentals of *R*Te_3_ layered tritelluride materials with a special focus on the recent advances made in electronic structure, CDW transition, superconductivity, magnetic properties, synthesis routes, and potential applications.

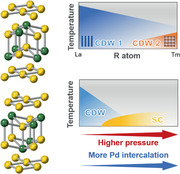

## 1 Introduction

After the discovery of graphene, ‐dimensional (2D) crystals have been one of the most popular research materials owing to their advanced optical, electrical, and magnetic properties that are not attainable in their 3D bulk counterparts or other traditional material systems.^[^ ^1^ ^]^ The order‐disorder phase transition is difficult to obtain in low dimensional materials because the interaction between microscopic constituents such as electrons and spins is not strong due to limited space. However, recently, the progress of quantum properties in 2D materials has grown swiftly. For instance, the existence of magnetism in 2D materials was not clear until the discovery of antiferromagnetic (AFM) FePS_3_ monolayers in 2016,^[^ ^2^, ^3^, ^4^ ^]^ and soon after in 2017 when ferromagnetism was discovered in monolayers of CrI_3_ and Cr_2_Ge_2_Te_6_.^[^ ^5^, ^6^ ^]^

Currently, emerging rare‐earth tellurides (*R*Te_3_) are layered materials that show a wide range of charge density wave (CDW) transition temperatures which can be tuned by changing rare‐earth elements and applying pressure. *R*Te_3_ with the heavy rare‐earth atom (*R* = Dy, Ho, Er, Tm) have two CDW transition temperatures,^[^ ^7^ ^]^ and they are observed to be superconducting (SC) by suppressing CDW^[^ ^8^, ^9^, ^10^, ^11^ ^]^ phases. GdTe_3_ has been reported to have the highest carrier mobility in the layered 2D AFM materials, and is comparable to black phosphorus.^[^ ^12^ ^]^ Other emergent quantum properties have been reported that suggest next‐generation quantum applications in the field. Here, we review the fundamental physical properties of the *R*Te_3_ system, emergent quantum phenomena arising from these unique quantum materials, and current synthesis methods to produce these materials.

## 2 Rare‐Earth Tritellurides

### 2.1 Crystal Structure

**Figure 1:**
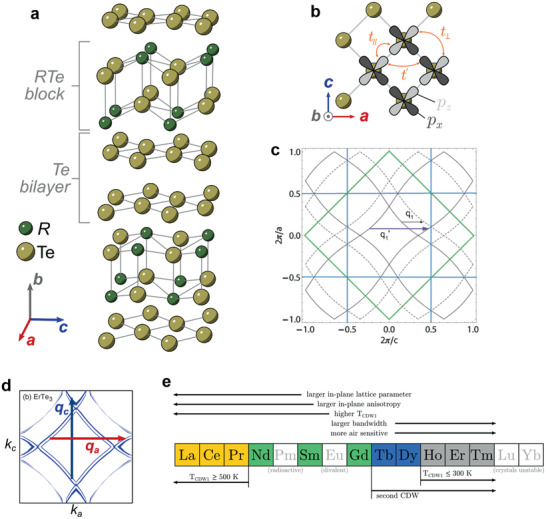
Crystal and electronic structure of *R*Te_3._ a) *R*Te_3_ structure consists of *R*Te block and Te bilayer. b) Top view of Te layer. The *p*_x_ and *p*_z_ orbitals are shown. c) Fermi surface in 2DBZ (green line) and 3DBZ (blue line). The arrow represents the CDW wavevector. Solid lines are Fermi surface in 2DBZ. Dotted lines are the Fermi surface folded by 3DBZ. d) Fermi surface of ErTe_3_ in 3DBZ calculated by DFT. e) Possible lanthanide elements that form *R*Te_3_ structure and the summary of trends across the lanthanide series. Figure 1a,b,e is reproduced with permission.^[^ ^19^ ^]^ Copyright 2020, Stanford University. Figure 1c is reproduced with permission.^[^ ^13^ ^]^ Copryight 2008, Stanford University. Figure 1d is reproduced with permission.^[^ ^20^ ^]^ Copyright 2008, American Physical Society.

The *R*Te_3_ family (*R* = Y, La, Ce, Pr, Nd, Sm, Gd, Tb, Dy, Ho, Er, and Tm) has a layered structure, and a single‐unit cell consists of two *R*‐Te slabs separated by two square Te sheets as shown in **Figure** **1**. Here, tellurium sheets are bonded weakly by van der Waals interactions which lend them their layered nature, and all *R*Te3 systems exhibit orthorhombic crystal structures with cmcm space symmetry. Cmcm symmetry has a glide plane between two Te sheets along the c‐axis direction but not along the a‐axis, and as a result, there exists a difference between the a‐ and c‐axis lattice constant which is small and non‐equivalent. For example, the c‐axis lattice parameter of TbTe_3_ is larger than a‐axis by 0.13% at room temperature.^[^ ^13^ ^]^ The difference between a‐ and c‐axis lattice parameters increases as the rare‐earth elements become lighter.^[^ ^14^ ^]^

Most rare‐earth elements form *R*Te_3_ layered structures (Figure 1a), and all *R*Te_3_ compounds have trivalent ion configurations. It is also noteworthy to mention that Pm from the lanthanide series is radioactive as such realizing PmTe_3_ is practically impossible even though Pm is likely to form PmTe_3_ from a chemical perspective. Similarly, the element Y is not technically in the lanthanides series, however, trivalent Y can also form an *R*Te_3_ structure with an ionic radius close to that of Ho, and thus YTe_3_ is also categorized within the lanthanide tritelluride series. While many of the lanthanide tritelluride series have been experimentally demonstrated to exist, EuTe_3_, YbTe_3,_ and LuTe_3_ have not been observed to date.

Since these layered crystals have their tellurene sheets and *R*Te sheets exposed to air, these material systems have lower environmental stability much similar to other 2D tellurium based material systems such as MoTe_2_, tellurene, and others.^[^ ^15^, ^16^, ^17^, ^18^ ^]^ In general, their stability decreases as the rare‐earth atoms become heavier as observed for TmTe_3_ in a 30‐min timeframe.^[^ ^19^ ^]^ In the materials, their degradation can be easily evidenced by optical imaging, Raman spectroscopy, and sometimes even by the naked eye as they change their color from a golden luster to a dull grey within a few hours.

In the *R*‐Te system, *R*_2_Te_5_ and *R*Te_2_ also consist of alternating Te sheets and *R*Te slabs. *R*Te_2_ remains stable at the highest temperature in these three structures while *R*Te_3_ is stable up to relatively low temperature. *R*_2_Te_5_ has *R*‐Te slabs separated by one and two Te sheets alternately. In *R*Te_2_, only one Te sheet exists between *R*‐Te slab layers and van der Waals interactions do not exist in this crystal system.

### 2.2 Electronic Structure

Since electronic conduction for *R*Te_3_ is dominated by Te sheets, we can obtain a good description of the electronic structure with a 2D unit cell of Te sheets, containing a single Te atom in the unit cell. The 2D unit cell is 1/$\sqrt{2}$ times the original 3D unit cell of the whole crystal structure and rotated by 45 degrees. Therefore, the Brillouin zone of the 2D unit cell (2DBZ) is $\sqrt{2}$ larger than that of the whole crystal structure (3DBZ) and rotated by 45 degrees. The electronic structure of *R*Te_3_ can then be described using a tight‐binding model of a tellurium layer due to its strong 2D character.^[^ ^21^, ^22^, ^23^ ^]^

Rare‐earth atoms ionize to *R* ^3+^ and Te atoms in *R*Te slab ionize to Te^2−^, which results in one electron donation to Te sheets. The neutral Te has the electronic configuration of 4*d* ^10^5*s* ^2^5*p* ^4^, and since electrons donated from the *R*Te slab are shared by two Te atoms in the adjacent Te sheets of *R*Te slab, the shared electrons fill *p* bands to 5*p* ^4.5^. This causes 5*p* ^4^ electrons to split due to the crystal's electric field and completely fill the out‐of‐plane 5*p*_y_ orbitals.^[^ ^24^ ^]^ The remaining *p* electrons are shared between 5*p*_x_ and 5*p*_z_ orbitals and form two orthogonal quasi‐1D bands. The *p*_x_ and *p*_z_ orbitals are shown in Figure1b, where *t*_∥_ represents the hopping along the extended p orbital, *t*_⊥_ represents the transverse hopping, and *t*′ represents the second‐nearest‐neighbor hopping, which is a combination of *p*_x_ and *p*_z_ orbitals. These hopping parameters have been estimated to be *t*_∥_ ≈ 2.0 eV, *t*_⊥_ ≈ 0.37 eV, and *t*′ ≈ 0.16 eV.^[^ ^24^, ^25^ ^]^ The experimental values are measured as *t*_∥_ ≈ 1.8 eV and *t*_⊥_ ≈ 0.35 eV.^[^ ^25^, ^26^ ^]^ Due to the wide band formed by *p*_x_ and *p*_z_ orbitals, *R*Te_3_ has a small effective mass and very high carrier mobility, and GdTe_3_ shows the highest mobility in all of the known 2D layered magnetic materials. Additionally, the mobility of GdTe_3_ is comparable to black phosphorus and is only surpassed by graphite.^[^ ^12^ ^]^ CDWs also contribute to high mobility because the scattering time can be improved by the gap opening due to the CDW.^[^ ^12^ ^]^

The resulting Fermi surface (of the entire crystal) as per the tight‐binding model of a Te sheet is translated into 3DBZ (Figure 1c,d). The Fermi surface shape is experimentally observed by positron annihilation^[^ ^27^ ^]^ and angle‐resolved photoemission spectroscopy (ARPES)^[^ ^25^, ^28^ ^]^ and the results are in good agreement with the expected Fermi surface shape and volume.

CDW is commonly observed in materials that have a low dimensional Fermi surface. When atoms move due to phonon interaction, electrons get arranged around positive nuclei such that the Coulomb potential is reduced. The induced negative charge is wavenumber dependent, and more charges can be induced at the wavenumber that connects two parallel portions of the Fermi surface. The wavevector associated with such a wavenumber corresponds to the CDW wavevector and is called the nesting vector. The phonon mode at this wavenumber is softened by the shielding effect. If the shielding effect and phonon softening at the nesting vector are large enough, then the atoms are fixed at the distorted position and form CDW. The new periodicity due to the distortion opens a gap at the Fermi surface, which lowers the energy of electrons at the Fermi surface. The CDW wavevector of *R*Te_3_ is described either by *q*_1_ ≈ (2/7)c* or $q_{1}^{\prime }\approx \left(5/7\right)c^{*}$ as shown in Figure 1c. They are identical due to the zone folding of 3DBZ. $q_{1}^{\prime }\approx \left(5/7\right)c^{*}$ nests on the 2DBZ Fermi surface while *q*_1_ ≈ (2/7)c* nests on one 2DBZ Fermi surface and one 3DBZ folded Fermi surface. In Figure 1c, solid lines represent the Fermi surface in 2DBZ, and dotted lines represent the Fermi surface folded by 3DBZ. $q_{1}^{\prime }\approx \left(5/7\right)c^{*}\;$is still valid even if there is no coupling between Te sheets and *R*Te slabs.

## 3 Physical Properties of Rare‐Earth Tritellurides

### 3.1 Charge Density Wave Phenomena in *R*Te_3_ Material Systems

To date, the CDW transition in *R*Te_3_ material systems has been observed by X‐ray diffraction (XRD),^[^ ^20^, ^29^, ^30^ ^]^ inelastic X‐ray scattering,^[^ ^31^ ^]^ electron diffraction,^[^ ^14^, ^21^, ^32^ ^]^ optical measurement,^[^ ^2^, ^33^, ^34^, ^35^, ^36^, ^37^, ^38^, ^39^ ^]^ ARPES,^[^ ^40^ ^]^ positron annihilation measurements,^[^ ^27^ ^],^ and electrical measurements.^[^ ^7^, ^20^, ^41^, ^42^, ^43^ ^]^

**Figure 2:**
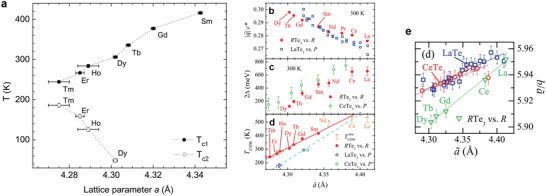
a) The CDW transition temperatures obtained from resistivity measurements, plotted as a function of in‐plane lattice parameters at 300 K. b) The magnitude of the CDW wave vector along c‐axis and c) the CDW gap 2Δ at 300 K for *R*Te_3_ series and LaTe_3_ and CeTe_3_ under applied pressure as a function of the average lattice constant $\tilde{a}$. d) The first CDW transition temperatures of *R*Te_3_ series and LaTe_3_ and CeTe_3_ under applied pressures. e) The ratio $\tilde{a}/b$ as a function of the average lattice constant $\tilde{a}$.*b* is the out‐of‐plane lattice constant. Figure 2a is reproduced with permission.^[^ ^20^ ^]^ Copyright 2008, Phys Rev B. Figure 2b–e is reproduced with permission.^[^ ^44^ ^]^ Copyright 2009, American Physical Society.

It is known that all *R*Te_3_ shows unidirectional incommensurate c‐axis CDW transition with wave vector *q*_c_ ≈ (2/7)c* at the transition temperature *T*_CDW1_. Tb‐Tm is observed to have a second a‐axis CDW transition at lower temperature *T*_CDW2_ with a wave vector *q*_a_ ≈ (1/3)a* perpendicular to *q*_c_ (Figure 1d). The unit cell volume of *R*Te_3_ changes with the atomic number of rare‐earth elements due to lanthanide contraction which creates internal chemical pressure. The CDW transition temperature *T*_CDW1_ changes with chemical pressure due to the variation of the density of states at the Fermi surface.^[^ ^20^ ^]^ As the atomic number increases, *T*_CDW1_ decreases from above 500 to 240 K. La‐Tb remains undistorted state at room temperature and the *T*_CDW1_ of Dy is just at room temperature (*T*_CDW1_ = 308 K) (**Figure** **2**a). The third CDW transition was revealed by optical conductivity measurements^[^ ^2^ ^]^ (**Table** **1**) and waiting to be observed by the diffraction measurements.

**Table 1:** Reproduced with permission.^[^ ^2^ ^]^ Copyright 2014, American Physical Society. CDW gap of the prior reported two transitions 2Δ_1_ and 2Δ_2_. The CDW gap of the third transition 2Δ_3_ reported by B. Hu et al.^[^ ^2^ ^]^ The transition temperatures were determined from the temperature dependence of electric resistivity. Reproduced with permission.^[^ ^20^ ^]^ Copyright 2008, American Physical Society. The transition temperatures of the third CDW have not yet been reported

	LaTe_3_	CeTe_3_	PrTe_3_	NdTe_3_	SmTe_3_	GdTe_3_	TbTe_3_	DyTe_3_	HoTe_3_	ErTe_3_	TmTe_3_
2Δ_1_ (10 K) [meV]	750	680	640	590	530	480	450	420	380	350	320
2Δ_2_ (10 K) [meV]	‐	‐	‐	‐	‐	‐	‐	50	90	110	140
2Δ_3_ (10 K) [meV]	370	350	320	310	290	270	260	250	‐	‐	‐
2Δ_1_ (300 K) [meV]	700	620	570	510	430	350	220	‐	‐	‐	‐
*T*_CDW1_ [K]	‐	‐	‐	‐	416	377	336	310	288	267	244
*T*_CDW2_ [K]	‐	‐	‐	‐	‐	‐	‐	52	110	157	180

The second bi‐directional CDW shows interesting pressure dependence. In most CDW materials, the increase of chemical pressure results in a reduction in the transition temperature. However, in *R*Te_3_, the increase of chemical pressure leads to an increase in the second transition temperature *T*_CDW2_. More specifically, *T*_CDW2_ increases from 50 to 190 K as chemical pressure increases from Dy to Tm (Figure 2a). This increase in *T*_CDW2_ is related to the reduction of *T*_CDW1_. Previously, ARPES experiments have revealed that in the compounds with smaller lattice parameters, the CDW gap is small around the Fermi surface.^[^ ^25^ ^]^ Therefore, the reduction of *T*_CDW1_ let more Fermi surface available for the second bidirectional CDW transition, which results in higher *T*_CDW2_.

Y. Chen et al. reported the thickness dependence of the CDW transition temperature of GdTe_3_ by Raman spectroscopy.^[^ ^45^ ^]^ The CDW transition temperature changed from 377 K for bulk to 431 K for 10 nm layer. This was attributed to chemical pressure by *R*Te slabs. Y. Chen et al. observed the blue shift of out‐of‐plane vibration of Te atoms as the thickness decreased, suggesting lattice compression along out‐of‐plane direction. The compression along out‐of‐plane causes the expansion of in‐plane Te sheets due to the Poisson effect. The expansion of Te sheets results in higher CDW transition temperature, which will be discussed in Section 3.1.1.

#### 3.1.1 Effect of Chemical Pressure and Applied Pressure on CDW

Sacchetti et al. have shown that the externally applied pressure affects the CDW properties similar to chemical pressure.^[^ ^44^ ^]^ The average in‐plane lattice constant $\tilde{a}=\;\left(a+c\right)/2$ was calculated and *R*Te_3_ (*R* = La‐Dy) and LaTe_3_ under applied pressure were compared. The magnitude of CDW modulation wave vector along c‐axis q at 300 K increases with decreasing average lattice constant for both chemical and applied pressure (Figure 2b). This similarity implies the modification of the Fermi surface and nesting properties under applied pressure. The comparison of the CDW gap 2Δ and the transition temperature *T*_CDW_ are shown in Figure 2c,d. From the data, the qualitative equivalence between chemical and applied pressure was observed. While 2Δ is almost identical considering its error bar, there was a small difference in *T*_CDW_ between these two types of pressure. They speculated that these behaviors are due to a difference in the effective dimensionality of the system. Chemical pressure is more 3D compared to applied pressure (Figure 2e).

Zucco et al. has reported the applied pressure dependence of two CDW transition temperatures of GdTe_3_, TbTe_3,_ and DyTe_3_. As mentioned by Sacchetti, the similarity between chemical and applied pressure was observed. The lower CDW transition temperature *T*_CDW2_ increased with applied pressure until it reached *T*_CDW1_ for TbTe_3_ and DyTe_3_. Although GdTe_3_ does not have the second transition temperature *T*_CDW2_ at ambient pressure, *T*_CDW2_ emerged by applying pressure above ≈1 GPa (Figure 6c). These transition temperatures were suppressed with applied pressure and SC states appeared at a lower temperature for these three compounds. SC states and suppression of CDW will be discussed in the later sections.

#### 3.1.2 Emergence of a New CDW Order by Light Excitation

**Figure 3:**
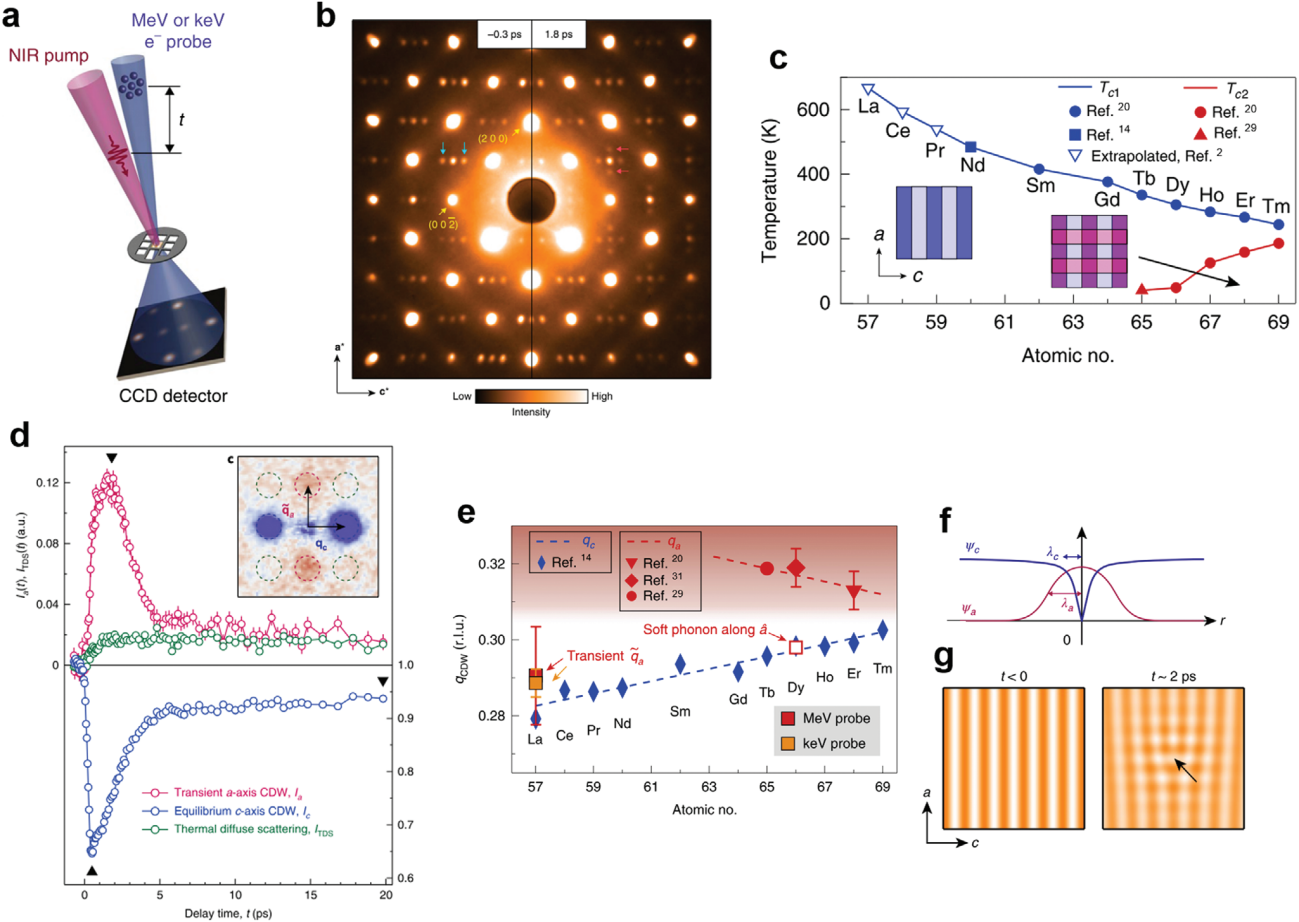
a) Schematic of the ultrafast electron diffraction setup in transmission mode. Reproduced with permission.^[^ ^53^ ^]^ Copyright 2019, Nature Publishing Group. b) Electron diffraction patterns before (left) and after 1.8 ps (right) NIR laser pulse excitation. Blue and red arrows indicate the equilibrium c‐axis CDW and the light‐induced transient a‐axis CDW peaks, respectively. c) CDW transition temperatures as a function of atomic numbers of rare‐earth elements. Blue dots indicate unidirectional CDW and red dots indicate bidirectional CDW. d) Time evolution of intensities of the light‐induced transient a‐axis CDW and the equilibrium c‐axis CDW and thermal diffuse scattering. e) The magnitude of CDW wavevectors as a function of atomic numbers of rare‐earth elements. The white square represents the calculated wavevector of the soft phonon along a‐axis confirmed by inelastic X‐ray scattering. Red and orange squares represent the wavevector of the a‐axis transient CDW observed by MeV and keV electron diffraction, respectively. f) Schematics of CDW order amplitudes, *ψ*_c_ and *ψ*_a_ near a topological defect in the equilibrium c‐axis CDW. The characteristic lengths of the suppression of *ψ*_c_ and the enhancement of *ψ*_a_ are shown as *λ*_c_ and *λ*_a_, respectively. g) Schematics of CDW in real space before (left) and after (right) laser pulse excitation. Laser‐induced dislocation (black arrow) is used as an example of a topological defect.

Recent studies have shown that strong light excitation can be used to give a rise to a broken‐symmetry phase in some materials.^[^ ^46^, ^47^, ^48^, ^49^, ^50^, ^51^ ^]^ Kogar et al. have found that light excitation breaks the translational symmetry of LaTe_3_ and causes a transient non‐equilibrium CDW along a‐axis. In their experiment, NIR pulsed laser was illuminated and transmission ultrafast electron diffraction patterns of (*h*0*l*) plane were taken before and after the pump laser. The schematics of the experimental setup is shown in **Figure** **3**a.

As LaTe_3_ only has a unidirectional CDW state in equilibrium, the electron diffraction pattern before the pump laser shows CDW peaks along the c‐axis (Figure 3b, left). After photoexcitation, the c‐axis CDW diffraction pattern weakened, and new CDW states along the a‐axis emerged (Figure 3b, right). The a‐axis CDW on LaTe_3_ does not exist in equilibrium (Figure 3c), and this non‐equilibrium CDW lasted only for a few picoseconds. The residual intensity after a long time is due to laser‐induced heating that caused a thermal occupation of phonons, which is shown as thermal diffuse scattering in Figure 3d. The relaxation of the new CDW along a‐axis and the reestablishment of the original CDW state happen in the same timescale. This implies the competition between the new non‐equilibrium CDW state and the original CDW.

The wavevector of the non‐equilibrium CDW state was not similar to that of other rare‐earth tritellurides along a‐axis (Figure 3e). *q*_a_ values of other rare‐earth tellurides were significantly larger than *q_c_* while the wavevector of transient CDW ${\tilde{q}}_{a}$ was smaller than its trend. Therefore, the transient CDW is not just an extension of equilibrium a‐axis CDW. From the similarity of ${\tilde{q}}_{a}$ in LaTe_3_ and anomalous wavevector in DyTe_3_, the origin of the anomalous wavevector was discussed from previous inelastic X‐ray scattering measurements and density functional theory calculations on DyTe_3_.^[^ ^31^ ^]^ In DyTe_3_, strong a‐axis CDW fluctuations are observed in the form of phonon softening when c‐axis CDW is formed. The wavevector of the fluctuations *q*_a, soft_ is close to that of c‐axis CDW. When a‐axis CDW was formed, the wavevector of a‐axis CDW has a larger value *q*_a_. At high temperatures, the Fermi surface is almost isotropic (*q*_a,soft_ ≈ *q*_c_). However, after c‐axis CDW is formed, parts of the Fermi surface is already gapped.^[^ ^52^ ^]^ Therefore, the nesting conditions changed and a‐ and c‐axis CDW have different wavevectors (*q*_a_ ≫ *q*_c_).

Kogar et al. proposed a picture where the non‐equilibrium CDW was caused due to the generation of topological defects excited by local absorption of high energy photons to explain the phenomena observed (Figure 3g). These defects were studied and characterized in LaTe_3_^[^ ^54^ ^]^ and Pd‐intercalated ErTe_3_^[^ ^55^ ^]^. In equilibrium, c‐axis CDW forbids a‐axis CDW in LaTe_3_. However, the presence of topological defects locally suppresses c‐axis CDW and a‐axis CDW appears. From this picture, anomalous transient wavevector ${\tilde{q}}_{a}$ of a‐axis CDW can be attributed to the absence of the c‐axis CDW at the defects. This picture also explains the origin of the timescale of relaxation of the transient a‐axis CDW and the reestablishment of equilibrium c‐axis CDW since the c‐axis CDW recovers and the transient a‐axis CDW cannot remain when the defects disappear.

To firm up the proposed mechanism, they performed a Ginzburg‐Landau analysis involving two complex order parameters, *ψ*_c_ and *ψ*_a_, which denote the equilibrium and transient CDW orders, respectively (Figure 3f). From the analysis, it was found that *ψ*_a_ is not zero around a defective core. Furthermore, they found that the characteristic length scale of the transient CDW *λ*_a_ extends longer than the defect core *λ*_c_. The ratio of *λ*_a_/*λ*_c_ is larger for less anisotropy between a‐ and c‐axis in the normal‐state. Therefore, the transient CDW is observable in wide‐area even though the defects are localized.

The CDW transition temperatures of *R*Te_3_ vary with *R* elements and can be tuned from low temperature to even above room temperature, which can be an advantage over other CDW materials for device applications. Although it is known that *R*Te_3_ compounds have two CDW transition and their properties are well studied, a new third CDW state (Table 1) and non‐equilibrium CDW states need to be further investigated.

### 3.2 Emergent Phenomena at High Pressures

#### 3.2.1 Introduction to High‐Pressure Science and Diamond Anvil Cell Design

**Figure 4:**
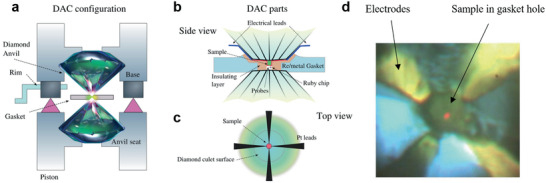
Introduction to DAC measurement. Reproduced with permission.^[^ ^56^ ^]^ Copyright 2020, Elsevier. a) DAC schematic. b,c) Side and top view of electrical DAC measurement. d) Optical image of in situ electrical DAC measurement.

The application of high pressure allows control of interatomic spacing and lattice parameters of materials while accessing optical, electrical, and magnetic properties in situ. Control over extreme pressures may induce structural and phase transitions, tune band structures, electrical transport properties, and even induce novel quantum properties such as superconductivity and CDWs. Diamond anvil cell (DAC) can help achieve extreme pressures from tens to hundreds of gigapascals (GPa) depending on the contact surface area. As shown in **Figure** **4**a, the DAC consists of two opposing diamonds, and the sample is placed between and compressed by diamonds. Ruby fluorescence serves as the pressure gauge to monitor the pressure inside DAC. With the advantage that diamond is an excellent transmitting material within a wide range of excitation wavelength, in situ optical characterization like XRD. photoluminescence (PL) and Raman spectroscopy are accessible.

#### 3.2.2 Electrical and Magnetic High‐Pressure Setup

Previous studies have focused on in situ electrical transport^[^ ^56^, ^57^ ^]^ and magnetic susceptibility^[^ ^58^, ^59^ ^]^ measurements on a variety of different *R*Te_3_ material systems at high pressures to capture emergent quantum phenomena and associated phase transitions. Figure 4b,c shows the schematic of electrical DAC measurement. Much similar to optical DAC configuration, the sample is placed in a gasket and compressed by two well‐aligned opposing diamonds. Here, the key to a successful electrical measurement required the insulation of the metallic gasket and the ability to make 4 electric probes in a much‐limited size DAC chamber. Generally, cubic boron nitride (c‐BN) powder serves as insulating materials and Au/Pt nanowire will be used as conducting leads. As shown in Figure 4d, four electrodes will be attached to the sample and the Au/Pt leads will extend out and connect to the copper wire using silver paste.

**Figure 5:**
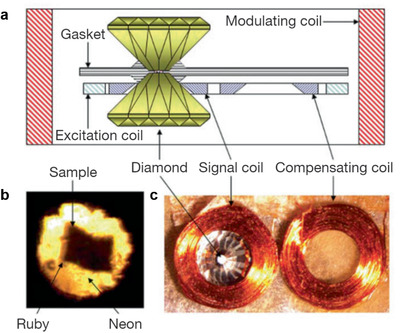
Magnetic susceptibility measurement in DAC measurement Reproduced with permission.^[^ ^60^ ^]^ Copyright 2010, Nature. a) Schematic of magnetic susceptibility measurement in DAC. b) The arrangement of sample, ruby, and pressure transmitting media under a microscope. c) Optical image for signal coil and compensating coil.

High‐pressure magnetic measurement in DAC is also developed in recent decades. In **Figure** **5**a, a schematic of a magnetic susceptibility measurement is shown. The DAC is designed from a Cu‐Be alloy to remove the ferromagnetic signal in steel DAC. For the same reason, conventional steel or rhenium gaskets are replaced with Ni‐Cr gaskets, and Neon or Helium are chosen as a pressure transmitting media to minimize the background magnetic signal. Similar to the vibrating sample magnetometer (VSM), the current will flow in the excitation coil and generate a magnetic field inside the DAC. The sample's response will be detected by the voltage change in the sensing/signal coil. Sample loading and coils arrangement is shown in Figure 5b,c.

#### 3.2.3 Pressure‐Induced Superconducting State in 2D Rare Earth Tri‐Tellurides

**Figure 6:**
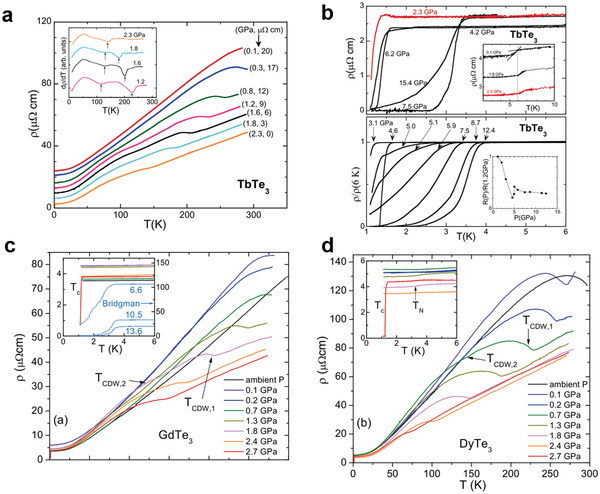
Pressure‐induced SC of TbTe_3_, GdTe_3_, and DyTe_3_. a,b) Electrical resistivity measurements at different pressures for TbTe_3_. c) GdTe_3_ and d) DyTe_3_. a, b) reproduced with permission.^[^ ^8^ ^]^ Copyright 2009, American Physical Society and c,d) reproduced with permission^[^ ^9^ ^]^ Copyright 2015, American Physical Society.

Motivated by the discovery of second charge wave density(CDW) transition in heavier rare‐earth (Dy, Tm) tri‐tellurides, Hamlin et al. in 2008^[^ ^62^ ^]^ assumed that the second CDW transition could be caused by rare‐earth substitution which can be understood as applying “chemical pressure”. Therefore, the studies were aimed at applying the “mechanical pressure” and studying the electrical transport in TbTe_3_ at high pressures. This seminal work has shown that a pressure‐induced SC state emerges at 2.3 GPa. Later in 2015, Zocco et al.^[^ ^62^ ^]^ focused on other *R*Te_3_ materials like GdTe_3_ and DyTe_3_, and they also conducted ac‐susceptibility experiments at a low‐pressure limit to determine the critical magnetic field. In **Figure** **6**a,b, electrical resistivity versus temperature is obtained at various pressures for TbTe_3_. From 0 to 2.3 GPa, no obvious drop in resistivity is observed down to 600 mK, the minimum of d*ρ*/d*T* is related to the first and second CDW transition. In Figure 6b, starting from 2.3 GPa, the electrical resistivity drops to immeasurable small at low temperature, indicating the SC transition. The transition temperature *T*_c_ increases with pressure ramping up and reaches nearly 4 K at 12.4 GPa. Further compression induced reduction in the transition temperature; therefore, the *T*_c_ may pass through a maximum within 12.4–15.4 GPa. Moreover, the upper inset of Figure 6b shows resistivity behavior near Neel temperature, and both AFM and SC resistive anomalies are present at 2.3 GPa. To conclude, the TbTe_3_ under pressure is likely a magnetically ordered superconductor where long‐range AFM coexists with SC and offers a great opportunity to study the interplay and coexistence of CDWs, AFM order, and superconductivity. In Figure 6c,d, resistivity measurements are collected for GdTe_3_ and DyTe_3_: Similar to the TbTe_3_ case, the first and second CDW transitions are visible at the low‐pressure region (0.1–0.7 GPa), and the *T*_CDW_ shifts to lower temperatures with pressure. For both GdTe_3_ and DyTe_3_, the electrical resistivity drops sharply starting from 2.7 GPa and the *T*c is around 1.3 and 1.45 K. With further compression, the onset *T*_c_ for GeTe_3_ shifts to the higher temperature and reaches a maximum of 3 K at 13.6 GPa. It's noticeable that both DyTe_3_ and GdTe_3_ are AFM and the AFM transition is observable in DyTe_3_.

#### 3.2.4 Determination of Critical Field for Superconducting State in 2D Rare Earth Tri‐Tellurides

**Figure 7:**
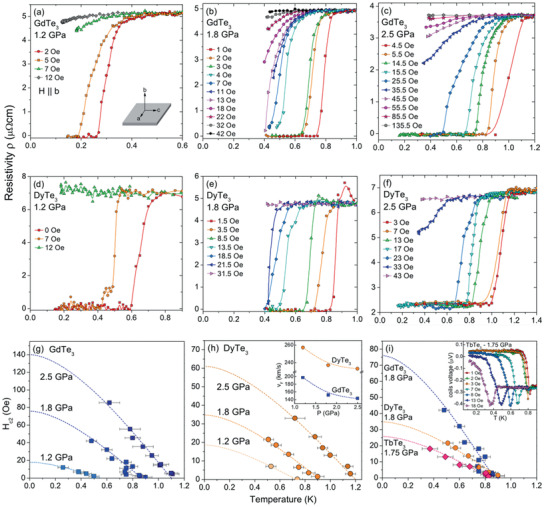
Reproduced with permission.^[^ ^9^ ^]^ Copyright 2015, American Physical Society. Determination of critical fields of GdTe_3_, TbTe_3_, and DyTe_3_ at different pressures obtained in the hydrostatic cell experiments. Magnetic fields were applied perpendicular to the planes; that is, *H* || *b* axis. a–c) Electrical resistivity of GdTe_3_. d–f) Electrical resistivity of DyTe_3_. g) *H_c_*_2_(*T*) of GdTe_3_ at 1.2, 1.8, and 2.5 GPa. h) *H_c_*_2_(*T*) of DyTe_3_ at 1.2, 1.8, and 2.5 GPa. The inset shows the evolution of the Fermi velocities *v*_F_ obtained from the slopes near *T*_c_. i) *H_c_*_2_(*T*) of GdTe_3_, TbTe_3_, and DyTe_3_ at similar pressures. For TbTe_3_, data were obtained from the ac‐susceptibility measurements displayed in the inset. The dashed lines plotted in (g–i) correspond to calculations of *H_c_*_2_(*T*) with the Werthamer, Helfland, and Hohenberg (WHH) model.

In addition to increasing the SC transition temperature in GdTe_3_ and DyTe_3_, Zocco et al. did further magnetic susceptibility investigation to determine the critical field *H*_c2_ for GdTe_3_, DyTe_3_, and TbTe_3_. In Figure 4a–c, ac susceptibility of GdTe_3_ is obtained in ^3^He–^4^He dilution refrigerator at 1.2, 1.8, and 2.5 GPa. Small magnetic fields are applied parallel to the b‐axis. At a lower temperature range, a smaller applied pressure is enough for GdTe_3_ to enter the SC state. The critical temperatures at different applied fields are plotted in **Figure** **7**g to show the relationship between critical field and temperature at different pressures. Similarly, ac susceptibility of DyTe_3_ is shown in Figure 7d–f and the extracted critical field *H*_c2_ versus temperature is plotted in Figure 7h. Last, the susceptibility of TbTe_3_ is shown in the inset of Figure 7i and the *H*_c2_(*T*) behavior for GdTe_3_, DyTe_3_, and TbTe_3_ are compared in Figure 7i. The results fit well with the Werthamer, Helfland and Hohenberg^[^ ^63^ ^]^ (WHH) model for a clean limit superconductor and the orbit limiting critical field can be calculated with formula $H_{c2}^{\mathrm{orb}}$= −0.73*T*_c_ $\frac{dHc2}{dT}|T_{c}$.

**Figure 8:**
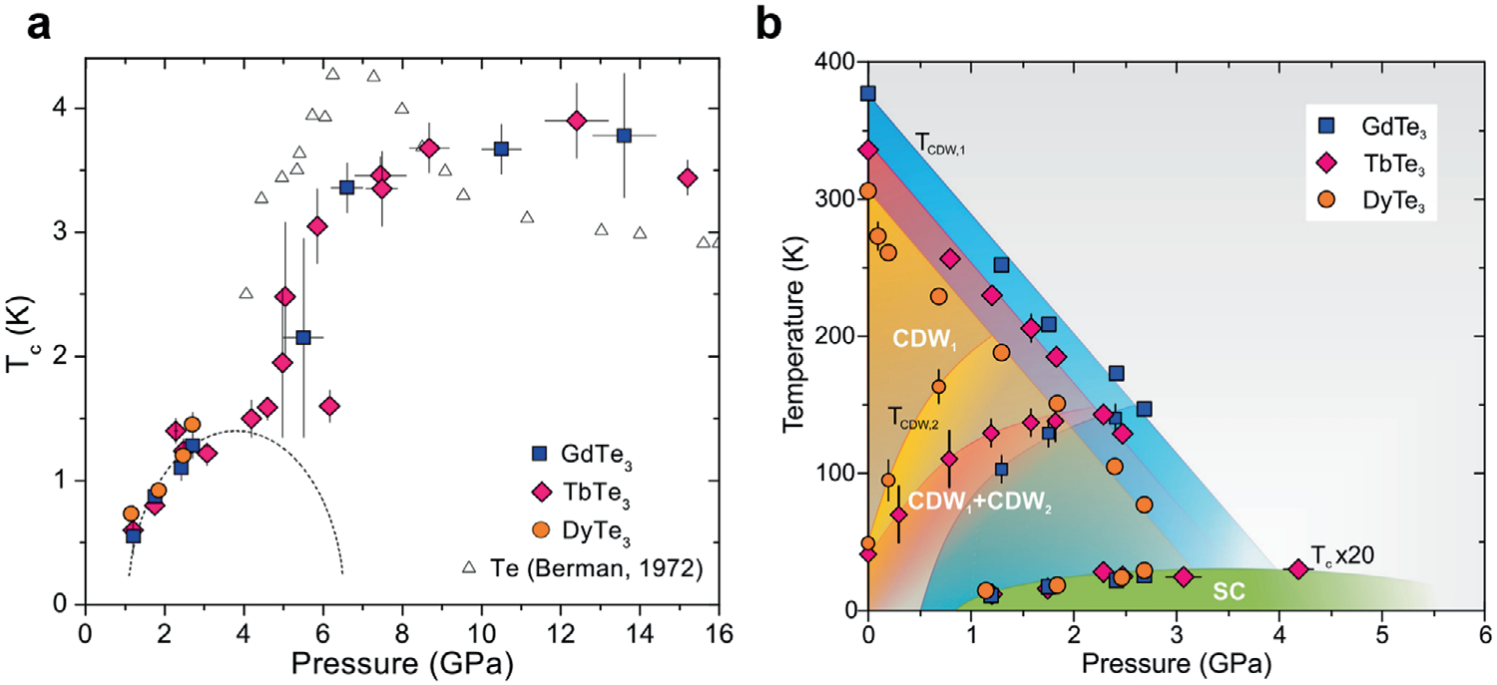
Reproduced with permission.^[^ ^9^ ^]^ Copyright 2015, American Physical Society. Pressure versus temperature phase diagram of GdTe_3_, TbTe_3_, and DyTe_3_. a) pressure dependency of onset temperature *T*_c_ of SC phase in GdTe_3_, TbTe_3_, and DyTe_3_. b) pressure versus temperature phase diagram of GdTe_3_, TbTe_3_, and DyTe_3_.

To summarize the pressure‐induced SC behavior of 2D rare earth tellurides, Zocca et al. plotted critical temperature versus pressure in **Figure** **8**a. The *T*_c_ is increasing monotonically at a low‐pressure range from 1 to 2.7 GPa and followed by a sharp jump from 1.5 to 3.5 K at around 5 GPa. The jump could be resulting from a structural transition where the lattice parameters and interatomic spacing will drastically change. With further compression, the *T*_c_ increases to a maxima around 4 K at 13 GPa and then reverses back. Another possible reason for the jump of *T*_c_ at 5 GPa may be the Te residue during the flux growth. Since Te itself becomes SC above 5 GPa and a similar jump is reported previously by Berman.^[^ ^64^ ^]^ The overlap and similarity of the SC phase of Te and *R*Te_3_ suggest the possibility that the jump could be caused by tellurium residue. To conclude the quantum states of 2D rare earth tellurides, a pressure versus temperature phase diagram is shown in Figure 8b, including two CDW states as well as the SC state of GdTe_3_, TbTe_3_, and DyTe_3_. The pressure‐induced SC provides new insight into 2D rare earth tellurides and the fundamental physics of interplay among CDW, AFM, and SC are unknown fields to study.

### 3.3 New Superconducting Phase by Topotactic Intercalation Process

Pd‐intercalated *R*Te*_n_* specimens are synthesized using solid‐state reaction techniques at moderate temperatures by repeatedly grinding together and heating product in a vacuum‐sealed quartz ampule to 500–700 °C for 50 h,^[^ ^10^ ^]^ or a Te self‐flux method at higher temperatures (800–900 °C) by adding Pd to the melted product and slowly cooling over 4 days.^[^ ^65^ ^]^ The detail of the synthesis methods will be discussed in Section 4.

**Figure 9:**
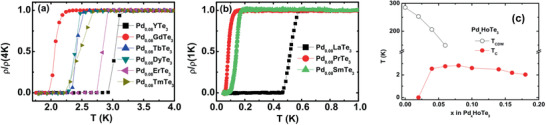
Reproduced with permission.^[^ ^10^ ^]^ Copyright 2016, IOP Publishing. Normalized temperature dependence of resistivity near the SC transition for polycrystalline Pd_0.8_ *R*Te_3_ compounds of various *R* compositions. In a) the resistivity is normalized to the resistivity at 4 K, showing SC transitions between 2–3 K. In b) the resistivity is normalized to 1 K, showing SC transitions from 1 to 0.6 K. c) CDW and SC transition temperatures as a function of Pd concentrations.

**Figure 10:**
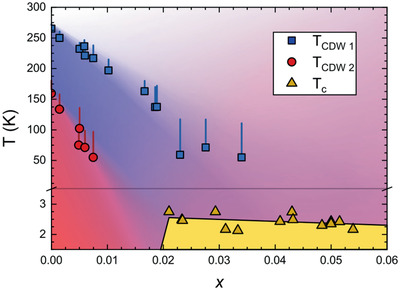
Reproduced with permission.^[^ ^11^ ^]^ Copyright 2019, American Physical Society. Electronic phase diagram of single crystal ErTe_3_ comparing the CDW temperatures and SC transition temperature (*T*_c_) as a function of Pd composition

The structure of *R*Te*_n_* typically consists of *R*Te slabs surrounded by sheets of Te oriented in the a–c plane (Figure 1a). In the case of Pd‐intercalation, the b‐axis (perpendicular to the orientation of the Te sheets) expands linearly with Pd concentration, suggesting that the Pd atoms reside in the van der Waals gap between the Te layers.^[^ ^11^ ^]^ These experimental results agree with predictions from DFT calculations.^[^ ^10^, ^65^ ^]^ Straquadine notes that at higher Pd concentrations (*x* > 0.035), the Pd begins to displace Te atoms and generate vacancies and that no crystalline material is achieved for *x* > 0.055. At standard pressures, superconductivity in both polycrystalline and single‐crystal *R*Te*_n_* structures emerges with Pd‐intercalation (Pd*_x_R*Te*_n_*) first reported by J. B. He et al. (2016).^[^ ^10^ ^]^ This SC property exists for compounds with both magnetic rare‐earth (Pr, Sm, Gd, Tb, Dy, Ho, Er, Tm) and nonmagnetic rare earth elements (Y, La), as shown in **Figure** **9**a,b. In polycrystalline material, the SC state is concentration‐dependent, appearing at low Pd concentrations *x* ≥ 0.02 and with a maximum *T*_c_ approaching 3 K near x = 0.08, and lower *T*_c_ at higher Pd concentrations (Figure 9c).^[^ ^10^ ^]^ Similarly, in crystalline Pd*_x_*ErTe_3_, the SC phase abruptly appears at *x* ≈ 0.02 and the *T*_c_ slightly decreases as Pd concentrations increase above *x* = 0.02, as shown in **Figure** **10**. Although the SC phases by applied pressure and disorder due to intercalation are similar, there were a few differences between them. The SC transition temperature after intercalation was almost constant above Pd concentrations of *x* = 0.02 while the transition temperatures by applied pressures increased up to 14 GPa. The maximum *T*_c_ values induced by Pd intercalation are lower than those observed at high pressures above 6 GPa.

The presence of both superconductivity and CDW in Pd‐intercalated rare earth makes this an interesting system to study the relationship between the CDW and SC states. It is expected that the CDW and SC state compete and that superconductivity would not exist until the CDW state is suppressed.^[^ ^66^ ^]^ As the Pd concentration increases, the CDW temperatures are suppressed, as shown in Figure 10. However, both the CDW and SC states coexist for 0.02 ≤ *x* ≤ 0.04 suggesting an alternative explanation is necessary.^[^ ^67^ ^]^

### 3.4 Magnetic Properties of Rare‐Earth Tritellurides

Recent advances in 2D magnetism have renewed interest in magnetic phenomena in any vdW crystals as well as 2D layers.^[^ ^5^, ^68^, ^69^ ^]^ Considering these new advances, we summarize the overall magnetic properties in these rare‐earth tritelluride systems. To date, the magnetic properties of *R*Te_3_ were investigated by magnetization and electrical resistivity measurements^[^ ^7^, ^13^, ^70^, ^71^, ^72^ ^]^ and heat capacity measurements.^[^ ^7^, ^13^, ^71^ ^]^ Since *R*Te_3_ consists of *R*Te slabs and Te sheets, the overall view is that *R*Te slabs contribute to magnetic properties whereas the individual Te sheets carry the electrical conduction. Since magnetic *R*Te slabs and conducting Te sheets are physically separated, the primary origin of magnetic interaction is the direct exchange or super‐exchange interaction instead of the RKKY interaction.^[^ ^70^ ^]^ The resistivity measurements also suggested that RKKY interaction is not significant, except in CeTe_3_.^[^ ^72^ ^]^.

YTe_3_ and LaTe_3_ which do not have f‐electron are nonmagnetic while the other *R*Te_3_ compounds show magnetic susceptibility.

#### 3.4.1 YTe_3_ and LaTe_3_

**Figure 11:**
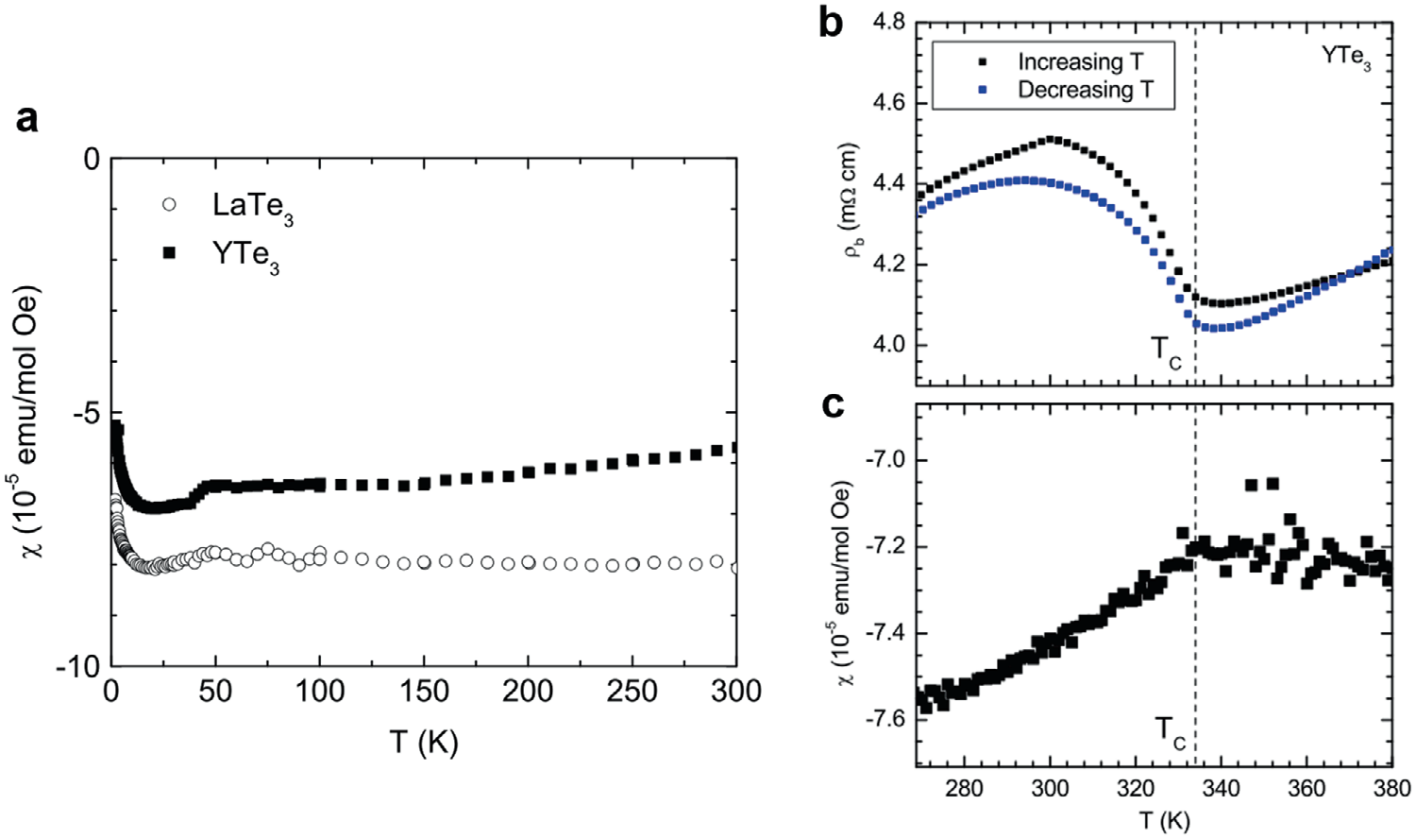
a) Magnetic susceptibility of LaTe_3_ and YTe_3_ along the in‐plane direction, Reproduced with permission.^[^ ^13^ ^]^ Copyright 2008, Starnford University. b) Resistivity of YTe_3_ along b‐axis. CDW transition was observed at *T*_c_. c) Magnetic susceptibility of YTe_3_ with *H*||*ac*. Figure 11b,c is Reproduced with permission.^[^ ^7^ ^]^ Copyright 2008, American Physical Society.

The susceptibility of YTe_3_ and LaTe_3_ is shown in **Figure** **11**a.^[^ ^72^ ^]^ These materials show temperature‐independent diamagnetic susceptibility. The small magnetic contribution observed at low temperatures arises due to the defects in these vdW crystals coming from low purity the rare‐earth element precursors (99.5%) which can be evidenced by a small feature at around 50 K due to trapped oxygen.

The temperature‐dependent resistivity and susceptibility in YTe_3_ are also shown in Figure 11b,c.^[^ ^7^ ^]^ The susceptibility decreases below the CDW transition temperature. This can be attributed to a smaller Pauli paramagnetic contribution due to the smaller density of states at the Fermi surface below the CDW transition temperature. In magnetic compounds, this feature is not obvious because the Curie susceptibility is large compared to Pauli paramagnetism. Here, we note that these measurements were mainly performed in the bulk form and their magnetic properties in the 2D limit currently remain largely unexplored.

**Figure 12:**
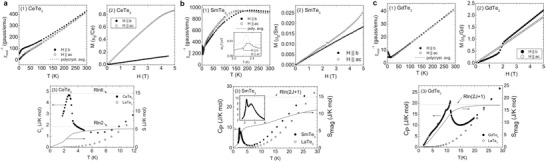
Reproduced with permission.^[^ ^13^ ^]^ Copyright 2008, Stanford University. a–c) Magnetic properties of *R*Te_3_ (*R* = Ce, Sm, and Gd). 1) The magnetic susceptibility as a function of temperatures for out‐of‐plane (*H*∥*b*) and in‐plane (*H*∥*ac*) direction. 2) Magnetization as a function of the magnetic field. 3) Specific heat of *R*Te_3_ (filled circles) and the magnetic entropy (solid line). The specific heat of the non‐magnetic LaTe_3_ is shown as a reference (open circles).

#### 3.4.2 *R*Te_3_ (*R* = Ce‐Nd, Sm, Gd‐Tm)

The magnetic susceptibility, the magnetization, and the heat capacity of *R*Te_3_ (*R* = Ce, Sm, Gd) are shown in **Figure** **12**a–c as examples.^[^ ^7^, ^13^, ^70^ ^]^ AFM transitions were observed in these compounds except for Pr, Er, and Tm. For Er and Tm, the magnetic transition was not observed down to 1.8 K.^[^ ^7^ ^]^ PrTe_3_ has a nonmagnetic singlet ground state due to the crystal field.^[^ ^13^, ^70^ ^]^ The magnetic transition temperature and magnetic parameters are summarized in **Table** **2**. The transition temperatures in Table 2 were obtained from heat capacity measurements since the transition temperatures can be observed clearly.

**Table 2:** Reproduced with permission.^[^ ^13^ ^]^ Copyright 2008, Stanford University. Magnetic parameters of *R*Te_3_ series. Neel temperatures *T*_N_ are determined from heat capacity. Weiss temperatures *θ* and effective moments *p*_eff_ from Curie‐Weiss fits are shown for *H*∥*b* and *H*⊥*b* directions and polycrystalline average. The values for Pr are from Ref 70; Reproduced with permission.^[^ ^70^ ^]^ Copyright 2003, American Physical Society. The calculated moments of rare‐earth ion $p_{R^{3+}}=\mu_{\mathrm{eff}}\;/\;\mu_{B}=g_{J}\;{\left[J(J+1)\right]}^{1/2}$ are shown in the last column

*R*	*T*_N_	*θ* ^∥^	*θ* ^⊥^	*θ* ^poly^	$p_{\mathrm{eff}}^{∥}$	$p_{\mathrm{eff}}^{⊥}$	$p_{\mathrm{eff}}^{\mathrm{poly}}$	$p_{R^{3+}}$
Ce	3.0	−30.0	1.58	−6.77	2.46	2.39	2.41	2.54
Pr	‐	(−23.7)	(12.0)		(3.68)	(3.67)		3.58
Nd	2.57, 2.64	−21.3	−3.01	−8.26	3.59	3.54	3.56	3.62
Sm	2.18, 2.6	−29.2	−7.92	−11.9	1.00	0.50	0.76	0.85
Gd	9.7, 11.3	−15.3	−15.3	−15.3	7.77	7.77	7.77	7.94
Tb	5.32, 5.51, 5.75	−24.0	−5.32	−9.66	10.1	9.83	9.88	9.72
Dy	3.44, 3.6	−6.60	−5.26	−5.26	10.8	10.5	10.6	10.63
Ho	2.92, 3.25	−0.64	7.84	−4.13	10.3	10.1	10.2	10.60
Er	< 1.8	−3.33	−0.19	−1.2	9.38	9.17	9.24	9.59

**Figure 13:**
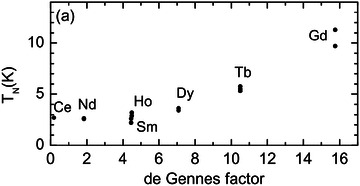
Reproduced with permission.^[^ ^7^ ^]^ Copyright 2008, American Physical Society. The Neel temperatures as a function of the de Gennes factor (*g_J_* − 1)^2^ *J*(*J* + 1).

The susceptibility of all these compounds followed Curie‐Weiss law, except SmTe_3,_ and the magnetic transition temperatures varied with rare‐earth elements. SmTe_3_ showed nonlinear susceptibility due to the small energy spacing between the ground state and the first excited state.^[^ ^73^ ^]^ The thermal excitation of Sm ion to the excited state results in the nonlinearity of inverse susceptibility. The relation between Neel temperatures and the de Gennes factor (*g_J_* − 1)^2^ *J*(*J* + 1), where *g* is the Lande factor and *J* is the total angular momentum, is shown in **Figure** **13**. The Neel temperature is expected to be proportional to the de Gennes factor.^[^ ^74^ ^]^ However, the experimental transition temperatures are not proportional to the de Gennes factor for *R*Te_3_. This discrepancy can be attributed to the effect of crystal electric field on the ground states of rare‐earth elements.^[^ ^7^, ^70^ ^]^

Most of the compounds except GdTe_3_ shows anisotropic magnetic properties between in‐plane and out‐of‐plane directions (Table 1). GdTe_3_ does not show anisotropy because the total orbital angular momentum of the ground state of Gd is *L* = 0 and the crystal electric field does not split the ground state.^[^ ^70^ ^]^ For most of the *R*Te_3_ compounds, the magnetization is larger in the in‐plane direction, which is called the easy axis.

## 4 Techniques for the Synthesis of Rare‐Earth Tritellurides

Currently, there are no reports of large‐scale, commercially viable synthesis methods, namely chemical vapor deposition (CVD) and atomic layer deposition (ALD) for producing few‐layer or monolayer rare earth tellurides discussed in this article. Environmental stability and a lack of understanding of the mechanisms responsible for layer‐layer deposition are the primary challenges to realizing the few‐layer or monolayers of these rare earth tellurides. To enable the investigation of these layered SC tellurides, bulk highly crystalline, defect‐free crystals must be produced. These crystals can then be mechanically exfoliated^[^ ^75^ ^]^ to study the layered nature of these novel tellurides. Literature reporting on the synthesis of rare earth tellurides is extremely limited but shows that melt‐phase flux zone growth and chemical vapor transport (CVT) are viable means of producing high‐quality defect‐free rare earth tellurides.

### 4.1 Flux Method

#### 4.1.1 Alkaline Metal Halides Flux

Early synthesis of *R*Te_3_ is achieved with flux growth using alkaline metal halides. DiMasi et al.^[^ ^76^ ^]^ used stoichiometric mixture of Sm and Te elemental precursors with RbCl and LiCl as flux. The reactant mixture was sealed in evacuated quartz ampules and heated up to 680 °C for 3 days. After slow cooling to 540 °C followed by fast cooling to room temperature, flux was rinsed away with water, ethanol, and acetone.

Using a similar method, the same group reported growth of HoTe_3_ and ErTe_3_ using KI as flux.^[^ ^21^ ^]^ Considering the melting point of TmTe_3_ (553 °C) which is readily lower than KI, LiI was chosen for the growth of this crystal. Later in 2003,^[^ ^72^ ^]^ Iyeiri et al. reported the successful synthesis of *R*Te_3_ (*R* = Ce, Pr, Nd, Gd, Dy) crystals using 1:1 RbCl and LiCl as flux, which resulted in rectangular reddish‐yellow crystal with size up to 3 × 3 × 0.1 mm.

We note that this method can also be used to grow *R*Te_2_ type of materials. DiMasi^[^ ^77^ ^]^ also reported growth of LaTe_2–_ *_x_*Sb*_x_* using RbCl and LiCl flux, with higher La concentration and elevated growth temperature.

#### 4.1.2 Self Flux

**Figure 14:**
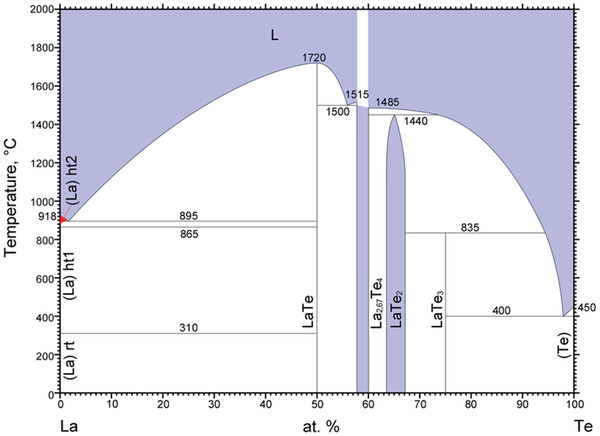
La‐Te binary phase diagram.

N. Ru et al.^[^ ^72^ ^]^ reported self‐flux technique using tellurium as a flux to synthesize *R*Te_3_ crystals (*R* = Y, La, and Ce). Compared with flux growth using alkaline metal halides, this approach avoided the introduction of defect elements in the crystal, reduced tellurium vacancies, and produced large crystals with good crystallinity. Taking LaTe_3_ as an example, the La‐Te phase diagram in **Figure** **14** clearly shows that by cooling a Te‐rich binary melt from below 835 °C to > 450 °C (above the melting temperature of Te), LaTe_3_ crystal can phase separate from tellurium melt and be separated by centrifugation. In this report,^[^ ^72^ ^]^ elemental precursors of *R_x_*Te_1–_ *_x_* (*x* = 0.015–0.03) were loaded in an alumina boat and sealed in an evacuated quartz ampule. After heating at 800–900^ ^°C to achieve homogeneous melt, the mixture was slowly cooled to 500–600 °C for 4 days. Melt was decanted with centrifuge while hot and solid crystals with dimensions up to 5 × 5 × 0.4 mm were collected.

With a similar method, the same group also reported growth of single‐crystal *R*Te_3_ where *R* = Pr,^[^ ^7^ ^]^ Nd,^[^ ^7^ ^]^ Sm,^[^ ^7^, ^20^ ^]^ Gd,^[^ ^7^, ^20^ ^]^ Tb,^[^ ^7^, ^20^ ^]^ Dy,^[^ ^7^, ^20^ ^]^ Ho,^[^ ^7^, ^20^ ^]^ Er,^[^ ^7^, ^20^ ^]^ and Tm.^[^ ^7^, ^20^ ^]^ The self‐flux technique has since become the most common practice to synthesize *R*Te_3_ single crystals. A similar growth process with a higher temperature and a greater amount of rare earth precursor can also be used to produce *R*Te_2_ materials.^[^ ^78^ ^]^

The addition of a small fraction of Pd in the Er‐Te self flux system can result in a Pd‐intercalated crystal.^[^ ^11^ ^]^ The authors observed that when *x* > 0.03 is added to the Pd*_x_*ErTe_3_ system, a byproduct of PdTe_2_ was also formed. Larger Er concentration and higher decanting temperature could suppress the formation of the PdTe_2_ phase.

### 4.2 Chemical Vapor Transport

CVT is a well‐established method to produce high‐quality single crystals of many layered material systems, including a few reports of rare earth tellurides.^[^ ^79^, ^80^, ^81^ ^]^ CVT transports and crystalizes precursor materials from a cold‐zone to a hot‐zone for endothermic reactions, or it transports them from a hot‐zone to a cold‐zone for exothermic reactions. To synthesize crystals using CVT, stoichiometric quantities of precursors must be evacuated and vacuum‐sealed in thick (≈ 2 mm) silica ampoules. Careful consideration of available binary and ternary phase diagrams are made to determine the thermal processing required for successful growth. High‐temperature processing generates large vapor pressures within the ampoule, which is why thick‐walled silica ampoules are preferred. To carry out the elemental transport in the ampoule, halides such as iodine and bromine are used and facilitate the growth of large single‐crystals.^[^ ^82^ ^]^

Although CVT has been an established method for bulk crystal growth of 2D materials, only one report to date has investigated the feasibility of *R*Te_3_ growth using vapor transport. Synthesis of Gd polytellurides was studied using iodine as a transport agent. It was shown that several alloy concentrations of Gd‐Te were easily accessed in addition to GdTe_3_ by tuning the growth temperature to achieve the desired alloy of Gd‐Te.^[^ ^83^ ^]^

## 5 Future Aspects

### 5.1 Potential Fundamental Research

Addressing certain prime issues of *R*Te_3_ pertaining to environmental stability by virtue of functionalization with minimal deterioration in promising properties would prove effective to tackle scalable thin‐film growth.^[^ ^13^ ^]^ Surface functionalization techniques are required to decorate the surface to prevent surface degradation without sacrificing the attractive properties of these 2D layers. More studies are needed to elucidate the role played by point defects (vacancies) in determining their environmental stability. If these materials are not stable in 2D form, surface encapsulation techniques (such as h‐BN encapsulation) must be explored.

Currently, these materials are mainly available from their bulk counterparts by mechanical exfoliation. ALD, molecular beam epitaxy, CVD, and other techniques must be established for their large area and ideally manufacturing compatible methods to successfully transition these materials from lab setting to industry landscape.

As the thickness is reduced from bulk crystals to the thin film, it is demonstrated that the CDW order will be enhanced and the transition temperature will be increased due to chemical pressure release. For a better understanding of CDW transition, the challenges associated with thin‐film growth requires attention, even if thin films are unstable. The discovery of new CDW or semiconducting phases could now be witnessed in equilibrium *R*Te_3_ crystal, which could not be found otherwise. Complex phase diagrams of *R*Te_3_ discloses the interplay of superconductivity, AFM, and CDW, on account of which the probable influence of spin fluctuations on magnetoresistance is unescapable.^[^ ^84^ ^]^ This stimulates the microscopic origin of the interesting linear magnetoresistance properties in *R*Te_3_ devices. Also, detailed conducive angle‐dependent magnetoresistance and Hall measurements are not explained for the majority of *R*Te_3_, for example, TbTe_3_ thin films.^[^ ^84^ ^]^ In parallel, it is much needed to understand how to tune and balance the CDW and SC behavior in these unique set of materials through straining, alloying, and thickness engineering approaches.

Another interesting area of research will be related to Moire superlattices and twistronics. Similar to discoveries made in superconductivity in graphene Moire lattices^[^ ^85^ ^]^ or Moire excitons in transition metal dichalcogenide Moire lattices,^[^ ^86^, ^87^ ^]^ new studies on Moire lattices using these *R*Te_3_ crystals will bring new quantum effects and functionalities to light.

Recently discovered 2D Janus or polar crystals have also opened our eyes to new opportunities enabled by the colossal E‐field induced within the layers.^[^ ^88^, ^89^, ^90^, ^91^, ^92^, ^93^, ^94^, ^95^ ^]^ It will be interesting to see how broken mirror symmetry influences the quantum properties of these 2D *R*Te_3_ sheets or how induced colossal E‐fields change the electronic, magnetic, and even optical properties of these materials systems.

### 5.2 Potential Applications

Easily accessible CDW states in these *R*Te_3_ materials near room temperature grants opportunity to control their electrical transport properties via external field at a low energy cost, similar to what has been shown in 2D VS_2_ and TaS_2_^[^ ^96^, ^97^ ^]^. M. Hossain et al. have comprehensively reviewed the potential quantum device applications for 2D CDW crystals.^[^ ^98^ ^]^ In this review, we would like to point out the unique advantages *R*Te_3_ materials have to offer in the applications.

#### 5.2.1 Supercapacitors

Higher performance supercapacitor requires electrodes with a large surface area, good conductivity, and high energy storage density. Although the layered nature of 2D materials perfectly meets the geometrical requirement, bandgap opening, and reduction of carrier mobility at the 2D limit forbids the application of general 2D materials in the field. GdTe_3_ materials, however, are recently shown to possess electron mobility beyond 60 000 cm^2^ V^−1^ s^−1^, which is the highest among all known 2D magnetics.^[^ ^12^ ^]^ Moreover, GdTe_3_ retains good conductivity in the exfoliated thin form, which makes it a potential candidate for the electrode of supercapacitors.

#### 5.2.2 Spintronics

In addition to the charge property of electrons, spintronic devices take control over the spin information stored in electron motions. The application of magnetic materials in these devices help to write and read information and improve endurance over other storage technologies. Highly conductive *R*Te_3_ with antimagnetic ordering also offers application possibility in spintronics. In magnetoresistive random access memory (MRAM) devices, for example, data storage relies on the magnetic anisotropy of the storage layer, while the read operation is performed by sensing the resistance difference in on and off state in the magnetoresistive device.^[^ ^99^ ^]^ Large magnetoresistance (MR) is in turn important in reading reliability in MRAM devices. Recently, Xing et al. reported a large magnetoresistance as high as 5600% at 1.8 K_._ Experimentally evident angle‐dependent magnetoresistance and hall measurements reveal obvious anisotropic non‐linear behavior at high temperatures and isotropic linear one at low temperatures. Highly conductive *R*Te_3_ is hence viable for high spintronic AFM devices such as random‐access memory devices, magnetic sensors, and hard drives.^[^ ^84^ ^]^

#### 5.2.3 Moire Electronics and Superconductivity

Weak van der Waals interaction between layers in 2D materials allows for an easy architecture of heterostructures. By adjusting the material identity, layer numbers, stacking geometry, etc., a wide range of physical properties were observed.^[^ ^100^ ^]^ Cao's work in 2018 on twisted angle graphene opened up new opportunities for 2D materials engineering via controlling the stacking twist angle between layers. An extra degree of periodicity allows for band structure engineering, symmetry modification, and observation of highly correlated states in material systems.^[^ ^86^, ^87^, ^101^ ^]^ As members of layered materials with intrinsic magnetic ordering, CDW, and gate‐tunable superconductive states without the use of any ionic‐liquid or chemical doping, *R*Te_3_ certainly finds its applications in magnetic twistronic devices.

## Conflict of Interest

The authors declare no conflict of interest.
